# A RANDOMIZED CONTROLLED TRIAL OF THE EVERYDAY MEMORY AND METACOGNITIVE INTERVENTION IN OLDER ADULTS

**DOI:** 10.1093/geroni/igad104.2618

**Published:** 2023-12-21

**Authors:** Ann Pearman, MacKenzie Hughes, Emily Giannotto, Clara Coblenz, Ethan Flurry, Taylor Curley, Christopher Hertzog

**Affiliations:** MetroHealth Medical System, Cleveland, Ohio, United States; Georgia Institute of Technology, Atlanta, Georgia, United States; Emory University School of Medicine, Atlanta, Georgia, United States; Georgia Institute of Technology, Atlanta, Georgia, United States; Mississippi State University, Starkville, Mississippi, United States; Air Force Research Laboratory, Dayton, Ohio, United States; Georgia Institute of Technology, Atlanta, Georgia, United States

## Abstract

This study evaluated an intervention to improve remembering in everyday life, the Everyday Memory and Metacognitive Intervention (EMMI). EMMI uses strategies designed to enhance everyday remembering and self-regulatory approaches to monitor goal pursuit, anticipate, and address memory demands. A randomized controlled trial compared a group assigned to EMMI with a traditional memory strategy training control (MSC) group. Expanding on earlier work (Pearman et al., 2020) we created a new event-based ecological momentary assessment (EB-EMA) smartphone application for reporting everyday memory errors when they occurred, synched to a nightly diary for collecting additional information about each event. The pretest-posttest design assessed changes in subjective memory beliefs and episodic memory performance and also measured posttest-only performance measures, including EB-EMA frequency of memory errors and a laboratory contact task assessing everyday prospective memory. The protocol was reduced in scope to accommodate fully remote participation due to the pandemic. The final sample included 30 EMMI and 32 MSC participants. Memory strategy training differentially improved MSC associative memory test performance. There were no group differences in EB-EMA reported memory errors or successful prospective memory contacts. The EMMI group showed more improvement in memory self-efficacy than the MSC group; both groups showed increases in perceived control over everyday memory. Individual differences in EMMI intervention benefits suggest that the program worked well in reducing memory errors for some but not all older adults. The EMMI shows promise but may require more extensive in-person training and behavioral shaping than this study provided to improve everyday memory.

